# Composition and Diversity of the Endobacteria and Ectobacteria of the Invasive Bark Beetle *Hylurgus ligniperda* (Fabricius) (Curculionidae: Scolytinae) in Newly Colonized Areas

**DOI:** 10.3390/insects15010012

**Published:** 2023-12-27

**Authors:** Ying Gu, Sixun Ge, Jiale Li, Lili Ren, Chuanzhen Wang, Youqing Luo

**Affiliations:** 1 Beijing Key Laboratory for Forest Pest Control, Beijing Forestry University, Beijing 100083, China; gu_ying@bjfu.edu.cn (Y.G.); gsx_pieris@bjfu.edu.cn (S.G.);; 2 Sino-France Joint Laboratory for Invasive Forest Pests in Eurasia, Beijing Forestry University, Beijing 100083, China; 3 Yantai Forest Resources Monitoring and Protection Service Center, Yantai 264000, China

**Keywords:** microbiota, invasion, organs, bark beetle, 16S rRNA

## Abstract

**Simple Summary:**

Symbiotic bacteria play an essential role in the digestion, detoxification, and nutrient supply of bark and ambrosia beetles. They may even promote the invasiveness and adaptability of invasive beetles. However, as a newly invasive pest in China, the diversity and community structure of bacteria associated with *Hylurgus ligniperda* is still unclear. This hinders our ability to comprehend their invasion mechanism and ecological adaptability. Therefore, this study revealed the differences in the diversity and community composition of associated bacteria in the beetle’s elytra, prothorax, and gut. Our findings revealed that the microbial population of the elytra was distinct from that of the prothorax and gut. Additionally, gender disparities existed throughout the bacterial population of elytra. The distribution of associated bacteria in different organs of males and females may indicate that they undertake different ecological functions. This study will help to understand the bacterial communities that may play important roles in *Hylurgus ligniperda*.

**Abstract:**

*Hylurgus ligniperda* (Fabricius) (Curculionidae: Scolytinae) is a new invasive pest beetle in China, which colonized the Shandong province, causing devastating damage. Originating in Europe, it has spread to Oceania, Asia, North and South America. Bacterial associates have been frequently reported to play a vital role in strengthening the ecological adaptations of bark and ambrosia beetles. The environmental adaptability of *H. ligniperda* may be supported by their associated bacteria. Bacterial communities colonizing different body parts of insects may have different functions. However, little is known about the bacteria associated with *H. ligniperda* and their potential involvement in facilitating the adaptation and invasion of the beetles into new environments. In this study, we employed high-throughput sequencing technology to analyze the bacterial communities associated with male and female adults of *H. ligniperda* by comparing those colonizing the elytra, prothorax, and gut. Results showed that the bacterial communities of male and female adults were similar, and the elytra samples had the highest bacterial diversity and richness, followed by the gut, while the prothorax had the lowest. The dominant phyla were Proteobacteria, Firmicutes, and Actinobacteriota, while the dominant genera were *Serratia*, *Lactococcus*, *Rhodococcus*, unclassified Enterobacteriaceae, and *Gordonia*. Among these, *Rhodococcus* and *Gordonia* were the specific genera of endobacteria and ectobacteria, respectively. Differences in the distribution of associated bacteria may suggest that they have different ecological functions for *H. ligniperda.* The results of functional prediction showed that bacteria were enriched in terpenoid backbone biosynthesis, degradation of aromatic compounds, limonene and pinene degradation, neomycin, kanamycin and gentamicin biosynthesis, indicating that they may assist their beetles in synthesizing pheromones, degrading toxic secondary metabolites of host trees, and antagonizing pathogenic fungi. These results help us understand the interaction between *H. ligniperda* and bacteria and highlight possible contributions to the invasion process.

## 1. Introduction

The red-haired pine bark beetle, *Hylurgus ligniperda* (Fabricius) (Coleoptera: Curculionidae: Scolytinae), is one of the most rapidly spreading invasive forest insects. *Hylurgus ligniperda* is considered native to Europe and the Mediterranean Basin but currently spread to all continents except Antarctica with the global trade in solid wood packing material, dunnage, or logs [1,2]. Many international and regional organizations have listed this insect as an important quarantine pest due to its ability to spread. In China, *H. ligniperda* was one of the most frequent forest pests in port quarantine, which was first discovered colonizing Yantai, Weihai, and Taian, Shandong Province, in 2019 [3,4,5].

*Hylurgus ligniperda* is a root- and stump-infesting beetle whose elytral declivity with reddish setae is strongly developed [6]. The beetle has a wide host range among conifer species, especially from the genus *Pinus* [5,7,8]. This beetle is considered a non-aggressive pest in Europe and the Mediterranean Basin due to its preference for ailing or dead trees [1,7]. However, *H. ligniperda* is multivoltine in tropical areas [7,9,10,11] and its life span can be up to 72 days in rearing conditions [12]. In addition, *H. ligniperda* has a strong fecundity, and its larvae and adults have similar habitats: a single female could lay up to 500 eggs [11] with the larvae and adults often feeding in a gallery together. Consequently, when they invade a new location, it may have disastrous impacts on the host plants. For example, *H. ligniperda* in Chile shows a very long life cycle and can infest throughout the year, resulting in the death of 10% of plantation seedlings [7]. *H. ligniperda* could also be an insect vector, carrying plant pathogenic fungi, such as *Alternaria* and the blue-stain fungi *Leptographium*, *Ophiostoma*, and *Sphaeropsi*. Studies on isolating fungi associated with *H. ligniperda* have been conducted in America, South Africa, Chile, Spain, and other countries [13,14,15,16,17,18]. For example, *Leptographium Procerum* (Ophiostomatales, Ascomycota), isolated from *H. ligniperda* in New Zealand, has been reported in China to have assisted the *Dendroctonus valens* (LeConte) (Coleoptera: Curculionidae: Scolytinae) in killing millions of pine trees [19]. In Europe and Northern Africa, the beetle is the nematode vector of the genus *Bursaphelenchus*, such as *B. hellenicus*, *B. sexdentati*, and *B. tusciae* [20,21,22]. As highlighted, the limited studies on the associations of *H. ligniperda* are much related to fungi and nematodes, but little is known about its bacterial associates and functions.

The bark beetles, whose niche is the phloem layer of trees, have to face the challenges of feeding on low-nutrient woody tissues and degrading toxic plant defense compounds [23,24,25]. Bark beetles significantly affect forest ecosystems due to microbial alliances supporting tree exploitation [26]. Several studies have shown that bacteria play important roles in bark beetle ecological success. The bacterial associates of bark beetles can contribute to their nutrition in three ways: (a) the promotion of fungal mutualists of mycophagous beetles, (b) the synthesis of nitrogen forms assimilable for beetles’ digestion, and (c) the hydrolysis of phloem tissue polymers to simple sugars [27,28,29,30,31,32,33,34]. Another aspect of the bacterial role in the successful occupation of beetles’ habitat is detoxifying the environment of the invaded host plant. Some bacterial species of genera *Pseudomonas*, *Serratia*, *Rahnella,* and *Erwinia* isolated from bark beetles have been shown to metabolize and reduce the concentrations of monoterpenes [35,36,37,38].

Bacteria are ubiquitous with bark beetles, and the ecological functions of associated bacteria are highly correlated with their colonization sites [39]. Symbionts providing digestive or detoxifying enzymes are consistently localized in the gut and related organs [40,41,42,43,44]. Additionally, those that defend their host or its nutritional resource from pathogens through competitive exclusion or the production of bioactive secondary metabolites are also localized in the gut or specialized cuticle-lined organs [45,46]. Moreover, some insects harbor symbionts on and in their body, which could benefit the host through nutrition supplementation [47,48,49,50]. Since the range of functions conferred by symbionts to their beetle hosts is reflected in their colonization sites, it is necessary to distinguish different body parts when studying insect-associated bacteria composition and potential function. Bacteria are also simply divided into endobacteria and ectobacteria according to whether they colonize inside or outside insect bodies [51]. Endobacteria refer to bacteria inside hosts extracellularly or intracellularly [52], including widely concerned gut bacteria, while ectobacteria refer to bacteria attached to the surface of mouthparts and cuticles [53]. Among them, bacteria within the mycangium of ambrosia beetles are particularly well known. However, we have not seen in literature or observed under microscope that *H. ligniperda* has mycangium. Therefore, the depressions on its body that can harbor bacteria could be good samples for studying ectobacteria.

The relationship between the strong adaptability of *H. ligniperda* to host trees and the function of their associated bacteria remains unclear. In this study, high-throughput sequencing based on the V3–V4 region in the 16S rRNA gene was used to investigate the associated bacterial communities of *H. ligniperda*. Specifically, we analyzed the community and possible ecological functions of the related bacteria colonized on three different body parts of *H. ligniperda*, i.e., elytra, prothorax, and gut. These results provide crucial empirical evidence for exploring the mechanisms behind the rapid population growth of this invasive species in China after colonization.

## 2. Materials and Methods

### 2.1. Sample Collection and Dissections

Newly emerged *H. ligniperda* were collected from the coastal protected forest near the Jiuguan Village in Muping District (Shandong Province, China) in August 2021 (37°27′21.21″ N, 121°52′27.74″ E). We used 40 traps (attractants with equal volumes of α-pinene and ethanol) in this forest dominated by *Pinus thunbergii* (Parlatore) (Pinaceae) and collected insects every two days. Samples from different traps were placed in separate 50 mL sterile microcentrifuge tubes using disinfected forceps. Healthy and active beetles were picked out and individually placed in 1.5 mL centrifuge tubes on the day of collection, stored at 4 °C. Within 3–8 days after collection, the samples were transported back to the quarantine laboratory (Beijing Forestry University) on ice and flash-frozen using liquid nitrogen, then stored at −80 °C until dissection.

**Figure 1:**
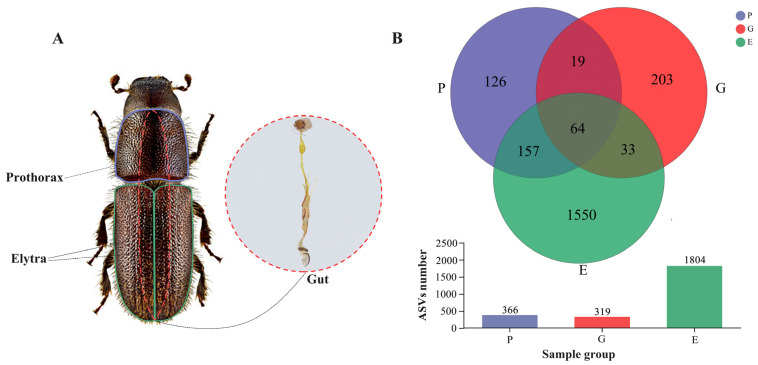
Schematic overview of *Hylurgus ligniperda* (male) samples and their associated ASV Venn diagram. (**A**) Schematic diagram of the three sampling sites of *H. ligniperda*: Prothorax, Elytra and Guts. (**B**) Venn diagrams of ASVs shared by three different colonization body parts samples of the *H. ligniperda*: P, Prothorax; E, Elytra; G, Guts.

Adults were fixed on a wax plate with sterile filter paper and carefully dissected using a dissection microscope under aseptic conditions. Sterilized dissection scissors were used to cut off the beetles’ elytra and prothorax to investigate ectobacteria, and fine-tipped forceps were used to pull out their intestines to study endobacteria. Then, anatomical samples were rinsed with 75% alcohol for 1 min to decrease contamination by environmental microorganisms or minimize cross-contamination between endobacteria and ectobacteria (especially intestinal and prothoracic samples). Subsequently, samples were rinsed three times with sterile water to eliminate residual alcohol. Although rinsing steps may result in the loss of some taxa of interest, it is necessary to remove transient taxa and optimize DNA extraction. After rinsing, samples were immediately placed into 1.5 mL centrifuge tubes containing 0.5 mL PBS and then flash-frozen with liquid nitrogen. Each of the three body parts (gut, elytra, and prothorax) was extracted and pooled into one sample from sets of 10 beetles, and five biological replicates were performed for each set of samples. So, 100 beetles (female = 50, male = 50) were used to make 30 samples (3 parts × 2 sexes × 5 replicates) in this study. Gender was identified by observing the presence (male) or absence (female) of the aedeagus on the dissected abdomen (Figure 1A) [54]. All samples were stored at −80 °C until processed for DNA extraction.

### 2.2. DNA Extraction, PCR Amplification, and 16S rRNA Gene Illumina Sequencing

The FastDNA@SPIN Kit for Soil (MP Biomedicals, Santa Ana, CA, USA) was used to extract the microbial community genomic DNA from elytra, prothorax, and gut samples of *H. ligniperda* following the manufacturer’s recommendations. Sample lysing was performed using FastPrep-24 5G grinder (MP, USA), oscillating at 6 m/s for 40 s. The DNA extract was checked on 1% agarose gel, and DNA concentration and purity were determined with a NanoDrop 2000 UV-vis spectrophotometer (Thermo Scientific, Wilmington, NC, USA). ABI GeneAmp^®^ 9700 PCR thermocycler (ABI, Carlsbad, CA, USA) was used to amplify the hypervariable region V3-V4 of the bacterial 16S rRNA gene using primer pairs 338F (5′-ACTCCTACGGGAGGCAGCAG-3′) and 806R (5′-GGACTACHVGGGTWTCTAAT-3′) [55]. Note: Although no negative controls were used during DNA extraction, several precautions were taken in order to minimize laboratory cross-contamination: all tools used were strictly sterilized and the entire process of DNA extraction was performed in an ultra-clean bench.

The PCR amplification of the 16S rRNA gene was performed as follows: initial denaturation at 95 °C for 3 min, followed by 27 cycles of denaturing at 95 °C for 30 s, annealing at 53 °C for 30 s and extension at 72 °C for 45 s, and single extension at 72 °C for 10 min, and end at 10 °C. The PCR mixtures contain 5 × TransStart FastPfu buffer 4 μL, 2.5 mM dNTPs 2 μL, forward primer (5 μM) 0.8 μL, reverse primer (5 μM) 0.8 μL, TransStart FastPfu DNA Polymerase 0.4 μL, BAS 0.2 μL, template DNA 10 ng, and finally, ddH_2_O up to 20 μL. PCR reactions were performed in triplicate. The AxyPrep DNA Gel Extraction Kit (Axygen Biosciences, Union City, CA, USA) was used to extract the PCR product from 2% agarose gel. The PCR product was purified according to the manufacturer’s instructions and was quantified using a Quantus™ Fluorometer (Promega, USA). Every set of amplifications contained negative controls (i.e., replacing the DNA template with an equal amount of water). Negative controls were not sequenced because no amplification product was observed in the gel.

According to the standard techniques of Majorbio Bio-Pharm Technology Co., Ltd. (Shanghai, China), purified amplicons were then pooled at equimolar concentrations and paired-end sequenced using the MiSeq PE300 platform (Illumina, San Diego, CA, USA). The raw reads were deposited into the NCBI Sequence Read Archive (SRA) database (Accession Number: PRJNA932973).

### 2.3. Sequence Data Processing

Fastp version 0.20.0 was used to filter the quality of the raw 16S rRNA gene sequencing reads [56]. The FLASH version 1.2.7 [57] was used to combine them with the following criteria: (i) the 300 bp reads were truncated at any site, receiving an average quality score of <20 over a 50 bp sliding window. The truncated reads shorter than 50 bp or containing ambiguous nucleotides were discarded. (ii) Only the overlapping sequences longer than 10 bp were assembled according to the overlapping sequence. (iii) The maximum mismatch ratio of overlapping regions was 0.2, and the reads that could not be assembled were discarded. (iv) Samples were distinguished by barcode and primers, and sequence direction was adjusted. A two-nucleotide mismatch was allowed in primer matching.

Amplicon sequence variants (ASVs) with a 100% similarity cutoff were clustered using DADA2 [58]. The chimeric sequences were identified and removed [59]. Classification analysis was performed on Quantitative Insights Into Microbial Ecology version 2022.2 (QIIME2) using a classify-sklearn (Naive Bayes) classifier trained against 16S rRNA database SILVA version 138 with a confidence threshold of 0.7 [60,61]. ASVs with a single sequence (singletons) and those identified as mitochondria or chloroplasts were excluded. These were classified as additional quality control or contaminants and removed prior to analysis. Many new synonyms have been identified in bacterial taxonomy (e.g., Actinobacteriota = Actinomycetota, Firmicutes = Bacillota, Proteobacteria = Pseudomonadota [62]). However, this study continued to employ the old taxonomic names since they are still frequently used, particularly in databases.

### 2.4. Statistical Analysis

The sequencing results were analyzed using the free online platform Majorbio Cloud Platform (https://cloud.majorbio.com/). Libraries were then rarefied to the same sequencing depth of 15,869 sequences and the rarefaction curve was used to assess whether the sequencing depth was sufficient and visualized with the “vegan” package in R v3.6.1 [63]. All subsequent analyses were conducted based on the rarefied data. The observed species richness (Sobs), Chao, ACE, Shannon, Simpson, Phylogenetic diversity (PD), Shannoneven, Simpsoneven and Coverage indices were calculated using mothur (version 1.30.2) to analyze community richness and diversity in samples [64]. All nine indices and the relative abundance of the top five phyla and top ten genera were compared between different groups with Kruskal–Wallis test by false discovery rate (FDR) (R package “stats”). Principal coordinates analysis (PCoA) at ASV level based on unweighted and weighted UniFrac distance metrics was estimated using QIIME (version 1.9.1, PERMANOVA, “adonis” function) [65] and visualized using R packages “vegan” and “ggplot2” [66] Then, a post hoc test was performed using Tukey–Kramer to find the sample groups with differences (R package “stats”). The “gplots” package in R was used to generate bar graphs based on bacterial composition and their relative abundance at phylum and genus levels. The Venn diagram visualizing ASVs that were shared or unique between three different body parts were plotted using the R package “stats”. Functional contributions of various taxa to different KEGG ortholog groups were computed with the “metagenome_contrib” command of PICRUSt2 [67] and visualized as heat maps.

## 3. Results

### 3.1. ASV Sequencing Results of the Prothorax, Elytra, and Gut

After quality filtering, a total of 1,311,131 high-quality sequences of 16S rRNA V3–V4 amplicon were generated from 30 samples to investigate the prothorax (P), elytra (E), and gut (G) bacterial communities of *H. ligniperda* (Supplementary Table S1). At 15,869 sequencing depth, a total of 2152 ASVs were detected, of which 64 were common within three groups, and 209 were shared between the two groups (i.e., P and G, P and E, and G and E). Compared to the 1550 unique ASVs detected in the elytra samples, only 126 and 203 unique ASVs were detected in the prothorax and gut samples, respectively (Figure 1). In total, 37 bacterial phyla, 78 classes, 191 orders, 333 families, 689 genera, and 929 species were identified (Supplementary Table S2). ASV-level rarefaction curves, which were used to evaluate sample richness and community uniformity, achieved a plateau, indicating that the sequencing depth of these samples was adequate. (Rarefaction curves: Supplementary Figure S1).

### 3.2. Bacterial Diversity Associated with the Prothorax, Elytra, and Gut of H. ligniperda

**Figure 2:**
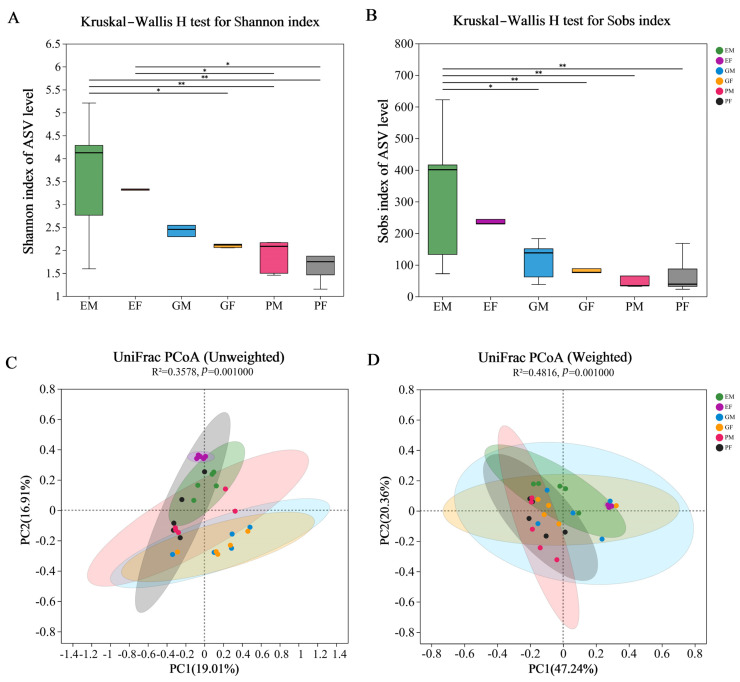
α–diversity and β–diversity of ASVs of three different body parts of the male and female *Hylurgus ligniperda*. (**A**) Species diversity (Shannon index). (**B**) Species richness (Sobs index). The significant differences of α−diversities were analyzed using the Kruskal–Wallis H test (* 0.01 < *p* < 0.05, ** 0.005 < *p* < 0.01). (**C**) Principal coordinate analysis based on unweighted Unifrac distances ASV. (**D**) Principal coordinate analysis based on weighted Unifrac distances. Ovals of different colors represent different groupings (adonis; *p* = 0.001). PF, the Prothorax of Female adults; PM, the Prothorax of Male adults; GF, the Guts of Female adults; GM, the Guts of Male adults; EF, the Elytra of Female adults; EM, the Elytra of Male adults. All of these abbreviations apply to the following figures.

Sobs, Chao, ACE, Shannon, Simpson, Phylogenetic diversity (Pd), Shannonven, Simpsoneven and Coverage indices were calculated to estimate bacterial diversity and richness associated with the *H. ligniperda*’s prothorax, elytra, and gut at the ASV level (Supplementary Table S3). None of the nine indices showed significant gender differences by Kruskal–Wallis test (Supplementary Table S4, *p* ≥ 0.1). According to the Shannon and Simpson indices, male samples for three body parts had greater values than female samples for the same body parts. According to all nine indices, bacterial diversity was the highest in the elytra, followed by gut, and the lowest in prothorax. Furthermore, the elytra exhibited significantly higher bacterial diversity than prothorax and gut based on the Sobs and Shannon index (*p* = 0.008 for Sobs index; *p* = 0.007 for Shannon index, Figure 2A,B).

PCoA based on unweighted and weighted UniFrac metric data was used to compare the similarities and differences of the bacterial community structure at ASV level in prothorax, elytra, and gut samples of both sexes. Under unweighted UniFrac, samples of both sexes from the same body parts were clustered together, while bacterial communities of different body parts clustered close but with significant differences (adonis, *R*^2^ = 0.3578, *p* = 0.001, Figure 2C). Specifically, the female elytra (EF) group was completely separated from the gut groups along the PC2 axis (Tukey–Kramer, Supplementary Table S5). However, under Weighted UniFrac, the bacterial community of female elytra clustered with two gut samples while separated from prothorax samples along the PC1 axis (adonis, *R*^2^ = 0.4816, *p* = 0.001, Figure 2D; Tukey–Kramer, Supplementary Table S6). These data suggested that most bacterial taxa associated with *H. ligniperda* were similar among different body areas, although each contained a certain number of unique bacterial species.

### 3.3. Bacterial Community Composition Associated with the Prothorax, Elytra, and Gut of H. ligniperda

**Figure 3:**
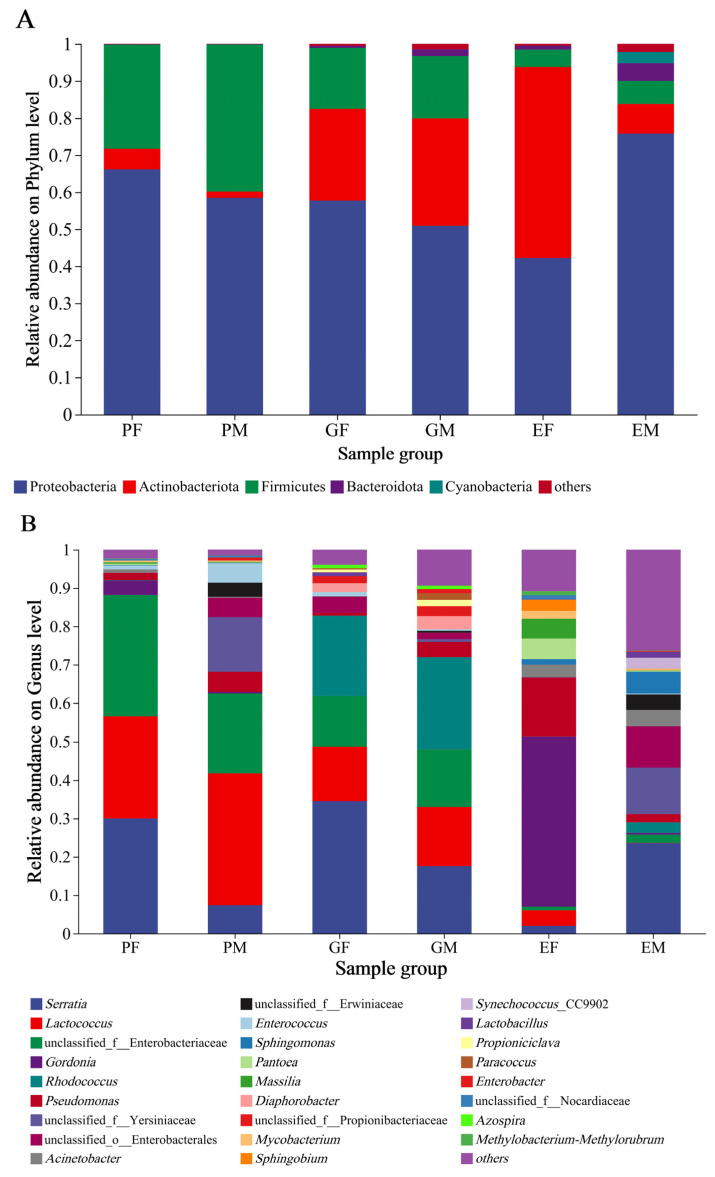
Relative abundance of bacterial phyla and genera of three different body parts of female and male *Hylurgus ligniperda*. (**A**) Relative abundance of dominant bacterial phyla (abundance ≥ 1%). (**B**) Relative abundance of dominant bacterial genera (abundance ≥ 1%). Different colors represent the relative percent abundance of bacterial genera. Species with abundance < 1% are denoted as “others”. PF, the Prothorax of Female adults; PM, the Prothorax of Male adults; GF, the Guts of Female adults; GM, the Guts of Male adults; EF, the Elytra of Female adults; EM, the Elytra of Male adults.

The community composition of bacteria associated with the prothorax, elytra, and gut of *H. ligniperda* was examined at phylum and genus levels (Figure 3). The relative abundance at the phylum level revealed that there were three dominant bacterial phyla detected in all *H. ligniperda* samples, with the male and female prothorax groups (PM, PF) showing similar composition (Proteobacteria 58.38%, 66.17%; Firmicutes 39.75%, 28.09%; Actinobacteriota 1.72%, 5.56% and Bacteroidota 0.07%, 0.08%). By contrast, in elytra groups, the male and female samples (EM, EF) were dominated by Proteobacteria (76.04%, 42.22%), Actinobacteriota (7.96%, 51.59%), Firmicutes (6.18%, 4.7%), Bacteroidota (4.79%, 1.06%), and Cyanobacteria (3.07%, 0). Similarly, the relative abundance of Actinobacteriota was increasing in the gut samples compared to the prothorax samples. The composition of bacterial communities associated with male and female samples (GM, GF) were Proteobacteria (50.91%, 57.72%), Actinobacteriota (29.08%, 24.77%), Firmicutes (16.79%, 16.45%), and Bacteroidota (1.78%, 0.56%) (Figure 3A).

At the genus level, 26 highly abundant genera (relative abundance > 1%) were detected in all samples (Figure 3B). In the prothorax group, males showed a more diverse dominant associated bacteria (eight genera): *Lactococcus* (34.47%), unclassified Enterobacteriaceae (20.72%), unclassified Yersiniaceae (14.28), *Serratia* (7.38%), *Pseudomonas* (5.23%), *Enterococcus* (5.04%), unclassified Enterobacterales (5.04%), and unclassified Erwiniaceae (3.70%), and females (six genera) were unclassified Enterobacteriaceae (31.63%), *Serratia* (30.01%), *Lactococcus* (26.54%), *Gordonia* (3.86%), *Pseudomonas* (1.79%), and *Enterococcus* (1.03%). Comparing with the prothorax groups, the gut-associated bacteria were with more abundant genera: in males (twelve genera), i.e., *Rhodococcus* (23.96%), *Serratia* (17.49%), *Lactococcus* (15.48%), unclassified Enterobacteriaceae (15.06%), *Pseudomonas* (4.00%), *Diaphorobacter* (3.46%), unclassified Propionibacteriaceae (2.56%), unclassified Enterobacterales (1.74%), *Paracoccus* (1.73%), *Propioniciclava* (1.66%), *Enterobacter* (1.10%), and *Azospira* (1.01%), while in females (eight genera) were *Serratia* (34.52%), *Rhodococcus* (20.98%), *Lactococcus* (14.20%), unclassified Enterobacteriaceae (13.13%), unclassified Enterobacterales (4.17%), *Diaphorobacter* (2.30%), unclassified Propionibacteriaceae (1.85%), and *Enterococcus* (1.14%). The predominant bacteria in the prothorax and stomach of males and females were highly similar. However, comparing with female samples, the overlap of bacterial genera in the elytra group was reduced in male samples (eleven genera): *Serratia* (23.64%), unclassified Yersiniaceae (12.12%), unclassified Enterobacterales (10.78%), *Sphingomonas* (5.71%), *Acinetobacter* (4.33%), unclassified Erwiniaceae (4.01%), *Synechococcus* (2.92%), *Rhodococcus* (2.78%), *Pseudomonas* (2.17%), unclassified Enterobacteriaceae (2.14%), and *Lactobacillus* (1.59%), while females (twelve genera) were *Gordonia* (44.45%), *Pseudomonas* (15.27%), *Pantoea* (5.32%), *Massilia* (5.25%), *Lactococcus* (4.11%), *Acinetobacter* (3.30%), *Sphingobium* (2.84%), *Mycobacterium* (2.05%), *Serratia* (1.98%), *Sphingomonas* (1.39%), unclassified Nocardiaceae (1.12%), and *Methylobacterium* (1.03%).

**Figure 4:**
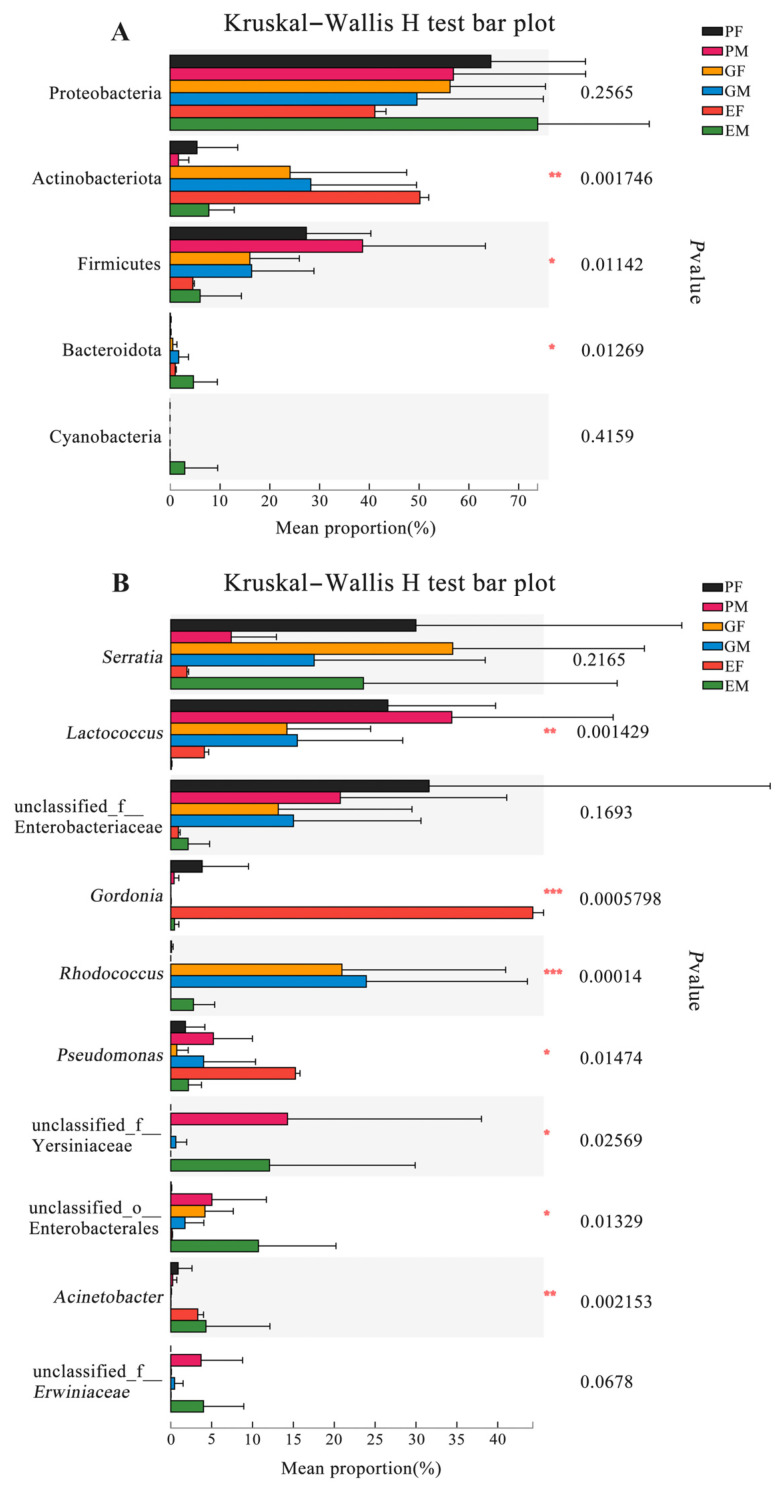
Abundance of dominant bacterial phyla and genera that colonized in different body parts of female and male *Hylurgus ligniperda*. (**A**) Differences in abundance of dominant bacterial phyla (the top five are shown). (**B**) Differences in abundance of dominant bacterial genera (the top ten are shown) (Kruskal–Wallis H test; * *p* < 0.05, ** *p* < 0.01, *** *p* < 0.001). PF, the Prothorax of Female adults; PM, the Prothorax of Male adults; GF, the Guts of Female adults; GM, the Guts of Male adults; EF, the Elytra of Female adults; EM, the Elytra of Male adults. Note: The error bar means standard deviation.

To further investigate the relative abundance differences of dominating bacterial phyla and genera in the gut, elytra, and prothorax samples of both sexes, we analyzed data from six groups using the Kruskal–Wallis test. Three of the five top abundant phyla (except Proteobacteria and Cyanobacteria) were significantly different among groups (Figure 4A) (Supplementary Table S7). Furthermore, among the most abundant ten genera, there were seven genera with significant differences between groups. *Lactococcus* (*p* = 0.0014) and unclassified Yersiniaceae (*p* = 0.0257) had the highest abundance in the male prothorax samples. *Gordonia* (*p* = 0.0006) and *Pseudomonas* (*p* = 0.0147) were the most abundant genera in the female elytra group, while unclassified Enterobacterales (*p* = 0.0133) and *Acinetobacter* (*p* = 0.0022) were the most abundant genera in the male elytra samples. *Rhodococcus* (*p* = 0.0001) was the most abundant one in the male gut samples (Figure 4B) (Supplementary Table S8).

### 3.4. Functional Predictions of the Endobacteria and Ectobacteria of H. ligniperda

**Figure 5:**
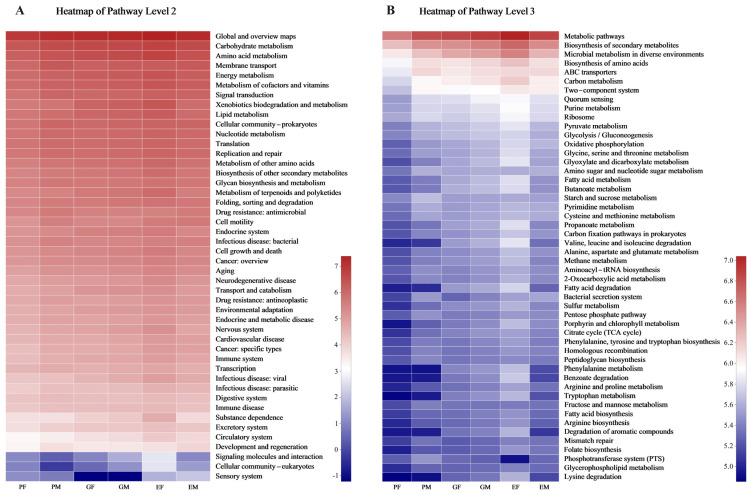
Prediction of KEGG functions of bacteria colonizing in different body parts of males and females (*Hylurgus ligniperda*). (**A**) Function prediction in pathway level 2. (**B**) Function prediction in pathway level 3. The top 50 pathways were listed and colors shifted from blue (lower) to red (higher) according to pathway abundance in each sample, indicating the logarithmic values of the relative abundances. PF, the Prothorax of Female adults; PM, the Prothorax of Male adults; GF, the Guts of Female adults; GM, the Guts of Male adults; EF, the Elytra of Female adults; EM, the Elytra of Male adults.

According to results generated by PICRUSt2 based on 16S rRNA gene sequences, the 46 level 2 KEGG pathways (Supplementary Table S9) and 394 level 3 KEGG pathways (Supplementary Table S10) were predicted for all samples. In pathway level 2, carbohydrate metabolism, amino acid metabolism, energy metabolism, membrane transport, cofactors, vitamin metabolism, xenobiotics biodegradation, and metabolism were enriched in all groups (Figure 5A). In pathway level 3, metabolic pathways, biosynthesis of secondary metabolites, microbial metabolism in diverse environments, biosynthesis of amino acids, and ABC transporters were shown as enriched in all groups (Figure 5B).

## 4. Discussion

This study describes the bacterial communities associated with *H. ligniperda*. This is the first survey focusing on the bacterial diversity and community structure of the endobacteria and ectobacteria of both sexes of *H. ligniperda*. Identifying variations in the bacteria associated with distinct body regions of each sex may help us comprehend the interactions between beetles and bacteria. Our results showed that bacterial diversity was generally not significantly different between males and females at different body parts. However, the bacterial diversity of the elytra samples was higher than that of the prothorax and gut samples. Furthermore, the bacterial composition of elytra varied between genders and was distinct from prothorax and gut. These bacteria, which develop a symbiotic association in different body sections of *H. ligniperda*, may improve its adaptability to host trees.

### 4.1. Diversity Differences of H. ligniperda-Associated Bacteria and Its Potential Causes

In our study, no significant difference was observed in the diversity of associated bacteria between both sexes at three body parts of *H. ligniperda*. This result was similar to previous works in other bark and ambrosia beetles [68,69,70]. The diversity of the elytra was the highest and their unique ASVs were also the most abundant, followed by the gut, and the prothorax was the lowest. The diversity of ectobacteria may be related to differences in surface area. As no mycangia, a structure that carries and stores symbiotic bacteria and fungi [8], have been observed in *H. ligniperda* at present, we initially hypothesized that puncta on the elytra and prothorax are harboring sites for associated bacteria [71,72,73]. The area of the elytra is larger than that of the prothorax, resulting in a greater number of puncta, which may be one of the reasons for the higher diversity and richness of associated bacteria on the elytra. However, the scanning electron microscope micrographs (Supplementary Figure S2) revealed the presence of yeast rather than bacteria in the puncta of the elytra, so the presence of abundant bacteria also could be due to the presence of obscure structures or mycangia on the elytra that have not yet been observed.

Under the unweighted unifrac, the EF samples completely separated from GF and GM samples, indicating that there might be many unique ASVs between the elytra and gut of *H. ligniperda*. This reconfirmed that sex was not a major influence factor on the bacterial community. While under the weighted unifrac, the EF samples were separated from the PF and PM samples, which indicated that the relative abundance of shared ASVs between the elytra and gut samples may be similar. In contrast, the bacterial communities of the EF and prothorax samples overlapped with those of ASVs that were less abundant. The effect of low abundance taxa was also present in the β-diversity analysis of the endomicrobiome and ectomicrobiome of *Dendroctonus simplex* (LeConte) (Coleoptera: Curculionidae: Scolytinae). Similar results often appeared in studies of the dynamic flora of some insects at different life stages [74,75].

### 4.2. Possible Ecological Role of High-Abundance Bacteria in the Invasion and Colonization of H. ligniperda

In our study, at the phyla level, Proteobacteria, Firmicutes, and Actinobacteriota dominated the *H. ligniperda* prothorax, elytra, and gut. Proteobacteria and Firmicutes have also been shown to be prominent in other bark beetles [76,77] and play important roles in promoting plant biomass digestion, supplementing nutrition, and degrading secondary metabolites from host plants [43,44,78,79]. Actinobacteriota isolated from other bark beetles has been reported to produce antimicrobial compounds and degrade cellulose [30,80]. Thus, these phyla with high abundance in and on *H. ligniperda* might also perform similar functions.

At the genus level, the composition of endobacteria and ectobacteria was somewhat different. In ectobacteria, *Gordonia*, *Pseudomonas,* unclassified Yersiniaceae, *Acinetobacter*, unclassified Erwiniaceae, *Sphingomonas*, *Pantoea*, *Massilia*, and *Mycobacterium* were abundant. The relative abundance of these genera on elytra was higher than prothorax. *Gordonia*, which serves as a dominant genus on female elytra of *H. ligniperda,* was also isolated from prothorax mycangia of ambrosia beetle *Platypus cylindrus* (Fabricius) (Coleoptera: Curculionidae: Platypodinae), but its role in the tree–beetle interaction remains unclear [81]. However, *Gordonia* isolated from the gut of *Periplaneta americana* (Linnaeus) (Blattodea: Blattidae: Blattinae) showed broad-spectrum antibacterial activity [82]. Notably, *Gordonia*, *Pseudomonas*, *Acinetobacter*, *Sphingomonas*, and *Massilia* were all reported to have a strong ability to biodegrade [41,83,84,85,86,87,88], while in endobacteria, *Rhodococcus*, *Diaphorobacter*, unclassified Propionibacteriaceae, *Propioniciclava*, *Azospira*, and *Paracoccus* were abundant. *Rhodococcus* of the phylum Actinobacteriota also have a high abundance in the gut of *D. valens* and they have been shown to be involved in the production of the pheromone verbenone [89]. Studies of the other unique genera of gut reported their roles in nitrogen transformation [90,91], suggesting that they might contribute to the nutrition of *H. ligniperda.*

*Serratia* and *Lactococcus* were highly abundant genera shared in and on the insect’s body. *Serratia* and *Lactococcus* isolated from *D. valens*’s gut have been reported to convert verbenol into verbenone [53], a pheromone that induces massive attacks. *Serratia* has been found to degrade the host defensive compounds α-pinene and d-pinitol [92], which greatly enhances the adaption to host trees. According to the results predicted via PICRUSt2, most of bacteria-associates with *H. ligniperda* were enriched in the biosynthesis of secondary metabolites, benzoate, limonene and pinene degradation, and neomycin, kanamycin, and gentamicin biosynthesis (Supplementary Table S10), indicating that they may assist *H. ligniperda* to synthesize pheromones, degrade toxic secondary metabolites of host trees, and antagonize pathogenic fungi.

### 4.3. The Limitations and Prospects of This Study

Insect gut bacteria are mainly influenced by diet composition [93], and environment in insect guts is relatively stable, thus ensuring a high degree of confidence in endobacteria results. In contrast, ectobacteria diversity is easily affected by sampling and the sample preparation process. Therefore, using insect traps to collect insects may not be the most suitable method as it may capture insects that are not typically associated with the ecological niche of *H. ligniperda*, thereby interfering with the results of ectobacteria. When studying surface bacteria on insects, it is recommended to obtain specimens directly from the environment where the insects reside, such as directly collecting *H. ligniperda* from galleries. Additionally, during the sample preparation process, we used 75% ethanol for disinfection for one minute. Undoubtedly, this step carries the possibility of removing certain target bacteria or reducing bacterial diversity. However, this step is necessary to minimize the impact of miscellaneous bacteria from the insect trap on surface bacteria analysis and avoid cross-contamination between surface and gut samples. Moreover, due to the lack of comparison with environmental samples, it remains unclear if the bacteria discussed here are stable symbionts or opportunists from the environment. Also, because samples from different developmental periods were not investigated, it was impossible to monitor the dynamic changes of the associated bacteria throughout the complete life cycle of *H. ligniperda*. As for associated bacterial function, even though predictions indicated that the associated bacteria of *H. ligniperda* may be involved in many metabolic pathways, the functions and capacities of different bacteria are very different. Many studies have also found that the physiological activity of bacteria also varies from strain to strain. Therefore, a large number of bioassays are needed to provide more direct evidence to clarify bacterial roles in beetle–host tree interaction in the future. We also will do further experiments on morphological observations to find the colonization sites of ectobacteria.

## 5. Conclusions

This study comprehensively analyzes the endobacteria and ectobacteria present in both males and females of the bark beetle *H. ligniperda* during the flight-raising period. Our investigation has clarified the highly abundant bacterial populations at the phylum and genus levels that colonize different body regions of *H. ligniperda*. Notably, we have observed that bacteria inhabiting the elytra exhibit greater diversity and a more complex composition. Furthermore, we have undertaken an initial exploration of the potential ecological roles assumed by these high-abundance associated bacteria, drawing on functional predictions from PICRUSt2 and prior research. Our findings highlighted the potential that these bacteria contribute to ecological functions such as detoxification, antagonism, and aggregation, which may enhance the beetle’s adaptability to diverse environments. This adaptability could be a contributing factor to its successful colonization across five continents. This research provides crucial empirical bacterial evidence for exploring the mechanisms behind the rapid population growth of this invasive species in China after colonization. However, it is imperative to acknowledge certain limitations in our study. The absence of comparisons with environmental samples prevents us from definitively establishing the source of these bacteria. Additionally, our investigation did not encompass samples from various developmental stages, precluding the monitoring of dynamic changes in associated bacterial communities throughout the complete life cycle of *H. ligniperda*. As a part of future research, we intend to conduct extensive bioassays to validate the ecological functions proposed in this paper.

## Data Availability

The datasets of 16S rRNA sequences for this study can be found in the NCBI Sequence Read Archive (SRA) database under accession number PRJNA932973. https://www.ncbi.nlm.nih.gov/bioproject/PRJNA932973/ (accessed on 26 December 2023).

## Supplementary Materials

The following supporting information can be downloaded at https://www.mdpi.com/article/10.3390/insects15010012/s1. Supplementary Figure S1: The Rarefaction curves of Sobs index on ASV level for each sample. (The sequencing depth at 15869); Supplementary Figure S2: SEM images of abaxial surface of the elytrum of *H. ligniperda*; Supplementary Table S1: Valid sequence statistics table of samples; Supplementary Table S2: Taxonomic information of bacterial communities in all samples; Supplementary Table S3: Diversity index at ASV level; Supplementary Table S4: Kruskal−Wallis test between groups for diversity indices at ASV level; Supplementary Table S5: Tukey−kramer test between groups for Beta diversity at ASV level (Unweighted UniFrac); Supplementary Table S6: Tukey−kramer test between groups for Beta diversity at ASV level (Weighted UniFrac); Supplementary Table S7: Kruskal−Wallis test of differences in abundance of associated bacteria between groups at the phylum level; Supplementary Table S8: Kruskal−Wallis test of differences in abundance of associated bacteria between groups at the genus level; Supplementary Table S9: Relative abundance of KEGG pathway Level 2; Supplementary Table S10: Relative abundance of KEGG pathway Level 3.
