# Supplementary material for: Composition and Diversity of the Endobacteria and Ectobacteria of the Invasive Bark Beetle Hylurgus ligniperda (Fabricius) (Curculionidae: Scolytinae) in Newly Colonized Areas
Source: Insects. 2023 Dec 27;15(1):12. doi: 10.3390/insects15010012 (PMC10815997; doi:10.3390/insects15010012)
Supplement: Supplementary file 1 [file insects-15-00012-s001.zip › Supplementary Figure S1.pdf]

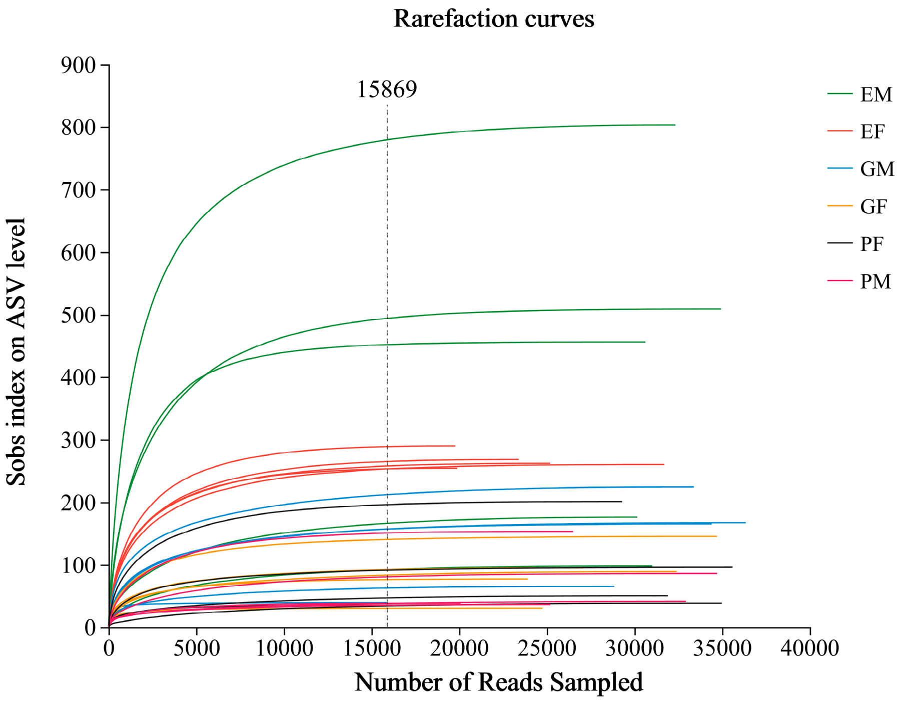

Figure S1. The Rarefaction curves of Sobs index on ASV level for each sample. (The sequencing depth at 15869). Note: PF,the Prothorax of Female; PM, the Prothorax of Male; GF, the Guts of Female; GM, the Guts of Male; EF, the Elytra of Female; EM, the Elytra of Male.
