# Supplementary material for: Composition and Diversity of the Endobacteria and Ectobacteria of the Invasive Bark Beetle Hylurgus ligniperda (Fabricius) (Curculionidae: Scolytinae) in Newly Colonized Areas
Source: Insects. 2023 Dec 27;15(1):12. doi: 10.3390/insects15010012 (PMC10815997; doi:10.3390/insects15010012)
Supplement: Supplementary file 1 [file insects-15-00012-s001.zip › Supplementary Figure S2.pdf]

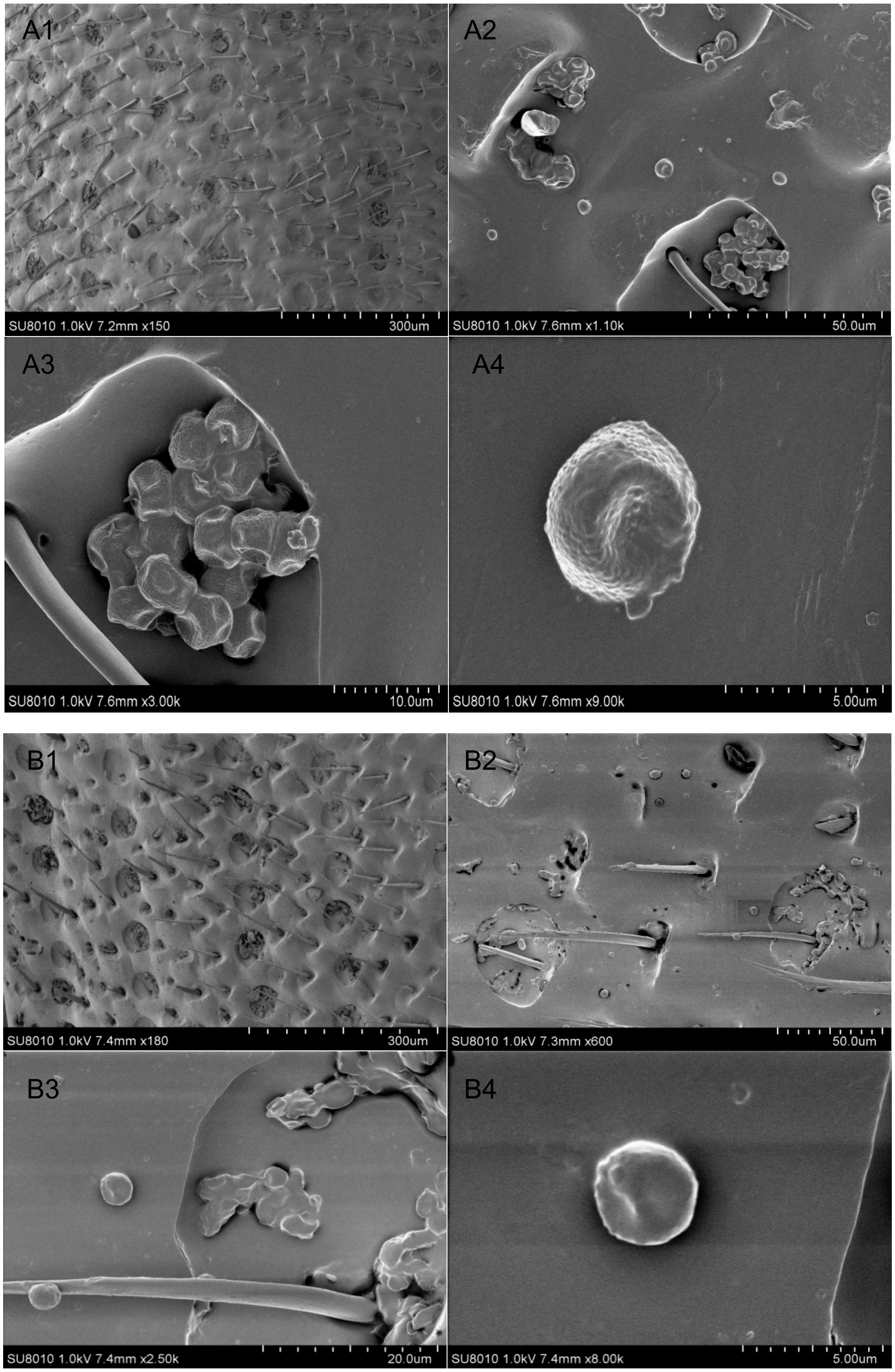

Figure S2. SEM images of abaxial surface of the elytrum of *H. ligniperda.* (A1-A4) SEM images of abaxial surface of the elytrum of *H. ligniperda* without surface disinfection. (B1-B4) SEM images of abaxial surface of the elytrum of *H. ligniperda* with surface disinfection.
